# 139D in NS1 Contributes to the Virulence of H5N6 Influenza Virus in Mice

**DOI:** 10.3389/fvets.2021.808234

**Published:** 2022-01-21

**Authors:** Kun Huang, Haiying Mao, Peilei Ren, Yufei Zhang, Xiaomei Sun, Zhong Zou, Meilin Jin

**Affiliations:** ^1^State Key Laboratory of Agricultural Microbiology, Huazhong Agricultural University, Wuhan, China; ^2^College of Veterinary Medicine, Huazhong Agricultural University, Wuhan, China; ^3^Key Laboratory of Development of Veterinary Diagnostic Products, Ministry of Agriculture, Wuhan, China

**Keywords:** H5N6, virulence, mice, NS1, 139D

## Abstract

H5N6, the highly pathogenic avian influenza A virus (IAV) of clade 2.3.4.4, causes global outbreaks in poultry. H5N6 has become the dominant IAV subtype in waterfowls and causes human infections with high mortality rates. Here, we isolated two strains of H5N6, XGD and JX, from chickens and ducks, respectively. Growth kinetics were evaluated in duck embryo fibroblasts, chicken embryo fibroblasts, Madin-Darby canine kidney cells, and A549 lung carcinoma cells. Receptor binding specificity was analyzed via sialic acid–binding activity assay. The virulence of each strain was tested in BALB/c mice, and recombinant viruses were constructed via reverse genetics to further analyze the pathogenicity. The two strains showed no significant differences in growth kinetics *in vitro*; however, JX was more virulent in mice than XGD. We also identified 13 mutations in six viral proteins of the two strains through genetic analysis. Our study showed that the NS1 protein played a crucial role in enhancing the virulence of JX. Specifically, the amino acid 139D in NS1 contributed to the high pathogenicity. Therefore, 139D in NS1 might provide insight into the underlying mechanism of IAV adaptation in mammals.

## Introduction

Influenza A virus (IAV) causes acute respiratory distress syndrome in many animal species. A novel H5N6 IAV has emerged via antigenic drift and reassortment events with other IAVs. This new highly pathogenic avian influenza (HPAI) A virus (subclade 2.3.4.4) has caused worldwide outbreaks in poultry (1). H5N6 was first isolated from mallards in 1975 (2). The novel recombinant H5N6 caused an outbreak in Laos in 2013 and was then isolated from waterfowls in southern China (3). H5N6 has replaced H5N1 as the dominant IAV subtypes in waterfowls that causes human infections in China (4). Therefore, the continuous evolution of the virus represents a long-term threat to public health and the poultry industry.

A wide range of avian species, including wild and domestic waterfowl, domestic poultry, and even zoo birds, are susceptible to H5N6 viruses. Compared with previous H5 HPAIs, the novel H5N6 virus exhibits altered pathogenicity in birds; H5N6 is lethal to chickens and ducks (5), even though ducks are a principal natural host of influenza A viruses, and influenza strains typically cause them no harm (6). Sixteen cases of human H5N6 infections have been reported since 2014 (7). A previous study showed that different H5N6 strains exhibit varying degrees of pathogenicity in mice (5, 7). Amino acid substitutions are associated with IAV virulence; for instance, the PB1 mutation D622G and the PB2 mutations E627K and D701N are closely related to replication and host tropism (8–10). However, the molecular mechanism underlying the high virulence of H5N6 in humans and mice remains unclear.

The non-structural protein 1 (NS1) of influenza A virus is an RNA-binding protein which antagonizes IFN-α/β mediated antiviral responses during influenza virus infection (11, 12). Therefore, it is an important internal factor that affects the virulence of several influenza A virus (13). For example, the single-amino-acid substitution of serine for proline at position 42 in the NS1 of H5N1 influenza virus antagonizes the host IFN response and increases the virulence in mice (14). Similarly, the five amino acid residues (EALQR) deletion of NS1 inhibits IFN induction and attenuate viral replication and virulence in mammalian cells and animals (15).

In this study, we investigated the pathogenicity of two H5N6 strains, A/duck/Hubei/WH18/2015 (JX; NCBI: txid1885579) and A/chicken/Hubei/XG18/2015 (XGD; NCBI: txid1885578), in BALB/c mice to elucidate the underlying mechanism of IAV adaptation in mammals. Our results showed that JX was more virulent than XGD. Genetic analysis revealed 13 mutations distributed among six proteins of JX and XGD. We also found that NS1 played a crucial role in enhancing the replication efficiency and virulence of JX in mice. Furthermore, we demonstrated that the amino acid 139D of NS1 in JX resulted in high pathogenicity. Further study of mutations like 139D is needed to clarify IAV mammalian adaptations and prevent future outbreaks.

## Materials and Methods

### Cells and Viral Strains

Duck embryo fibroblasts (DEF), chicken embryo fibroblasts (CEF), and Madin-Darby canine kidney (MDCK) cells were cultured in Dulbecco's modified Eagle's medium (HyClone, Logan, UT, USA) supplemented with 10% fetal bovine serum (PAN-Biotech, Aidenbach, Germany) and 100 U ml^−1^ penicillin-streptomycin (Thermo Fisher Scientific, Waltham, MA, USA). Lung carcinoma cells (A549) and human embryonic kidney cells (293T) were cultured in F12 medium (HyClone) and RPMI 1640 medium (HyClone), respectively. The H5N6 IAV strains A/duck/Hubei/WH18/2015 (JX) and A/chicken/Hubei/XG18/2015 (XGD) were isolated from ducks and chickens, respectively, and grown in 9-day-old embryonated eggs. IAV titers were established by determining the 50% tissue culture infective dose (TCID_50_) in MDCK cells.

### Growth Kinetics

The two strains were inoculated in DEF, CEF, A549, and MDCK cells monolayers cultured in 12-well plates, with a multiplicity of infection (MOI) of 0.01. Triplicate samples were established for each time point. The supernatants were collected at 12, 24, 36, 48, 60, and 72 h post-inoculation (hpi). The samples were then titrated on MDCK cells cultured in 96-well plates to calculate the TCID_50_ using the Reed and Muench method (16).

### Sialic Acid–Binding Activity Assay

To assess H5N6 receptor-binding specificity, a solid-phase enzyme-linked assay for influenza virus receptor-binding activity was performed as described previously (17). Purified viruses diluted with phosphate-buffered saline (PBS) to a hemagglutination titer of 1:20 were allowed to bind to the wells of fetuin-coated polyvinyl chloride enzyme immunoassay microplates overnight at 4°C. The plates were washed with 0.01 % Tween 80 in PBS (PBS-T) to remove unbound viruses. Serial 2-fold dilutions (0.625–10 μg mL^−1^) of Neu5Aca2-3Galb1-4GlcNAcb-PAA-biotin (3'SLN) and 6'-sialyl lacNAc-PAA-biotin (6'SLN) (GlycoTech, Gaithersburg, MD, USA) were added to the wells and incubated for 2 h at 4°C. After washing, horseradish peroxidase activity was assayed with o-phenylenediamine substrate solution. The absorbance at 630 nm was determined using a Tecan Spark 10 M multimode microplate reader (Tecan, Mannedorf, Switzerland).

### Assessment of Pathogenicity of JX and XGD Strains in Mice

Female BALB/c mice, 4–6 weeks of age, were purchased from the Center for Animal Disease Control, Hubei Province, China. To determine the 50% mouse lethal dose (MLD_50_) of the viruses, five mice were infected intranasally with 10^2^-10^6^ TCID_50_ of JX or XGD diluted in 50 μL PBS. The infected mice were observed for 14 d post-infection (dpi) to monitor weight loss and survival. All mice showing more than 20% body weight loss and respiratory distress were humanely euthanized. Groups of six randomly selected mice were intranasally administered 10^5^ TCID_50_ JX, 10^5^ TCID_50_ XGD, or PBS (control) and three mice was sacrificed at 3 dpi or 5 dpi to collect the lungs and heart for further study. For histopathological analysis, the hearts and the left hemisphere of each lung were fixed in formalin. For viral load determination, the right hemisphere of each lung was placed in 1 mL PBS containing 100 U mL^−1^ penicillin-streptomycin).

### Histopathology and Immunohistochemistry

Lungs were collected at 5 dpi and they were fixed in 10% neutral buffered formalin. Sections (4 mm thick) were stained with hematoxylin and eosin (H&E) and examined through light microscopy. For immunohistochemistry, Deparaffinized, rehydrated tissue sections were incubated in antigen retrieval buffer for 15 min at 97°C and endogenous peroxidase was quenched using 3% H_2_O_2_ in methanol for 10 min (18). Then, tissue sections were stained with rabbit-anti-NP antibody (GeneTex, USA, GTX125989, 1:200). Goat antirabbit immunoglobulin conjugated to peroxidase (Maxim Bio, Fujian, China) was used as secondary antibody. Screening of sections was performed with an Olympus BX51 microscope coupled to a camera.

### Pathogenicity of Recombinant Viruses

To generate recombinant viruses, we used an eight-plasmid reverse genetics system pHW2000. The *Bsm*BI sites were used to clone the cDNA of eight segments from JX and XGD H5N6 influenza virus. They were, respectively, cloned into the pHW2000 (19). The mutant viruses were rescued on the background of JX. Confluent 293T cells in six-well plates were transfected with 2 μg DNA of all plasmids encoding the eight viral segments, using Opti-MEM and Lipofectamine 2000 (Thermo Fisher Scientific). The medium was removed at 8 h post-transfection and replaced with fresh DMEM supplemented with 1% penicillin/streptomycin. At 48 h post-transfection, supernatants were harvested and inoculated in 9-day-old embryonated eggs, resulting in the rJX, rXGD, JX-PB1 (XGD), JX-PB2 (XGD)… and rJX-M (XGD) recombinant viruses. All the recombinant viruses were verified by sequencing and titrated based on the TCID_50_. The rJX-NS1-D139N/G211R recombinant viruses were rescued according to the above method. Groups of mice (*n* = 5) were intranasally infected with 10^3.7^ TCID_50_ of recombinant viruses. The infected mice were observed to monitor weight loss and survival for 14 days post-infection.

### Biosafety

All experiments involving live viruses were performed in a biosafety level 3 (BSL3) facility in accordance with the institutional biosafety manual. The animals were housed in negative pressure isolators with high-efficiency particulate air filters in the BSL3 facility.

### Statistical Analyses

Data are presented as the mean ± standard deviation of triplicate experiments. Groups were compared using an independent sample *t*-test or one-way analysis of variance (ANOVA) of GraphPad Prism (San Diego, CA, USA). The level of significance was set at *p* < 0.05. Two-way ANOVA was used when appropriate.

## Results

### Growth Kinetics of XGD and JX

**Figure 1:**
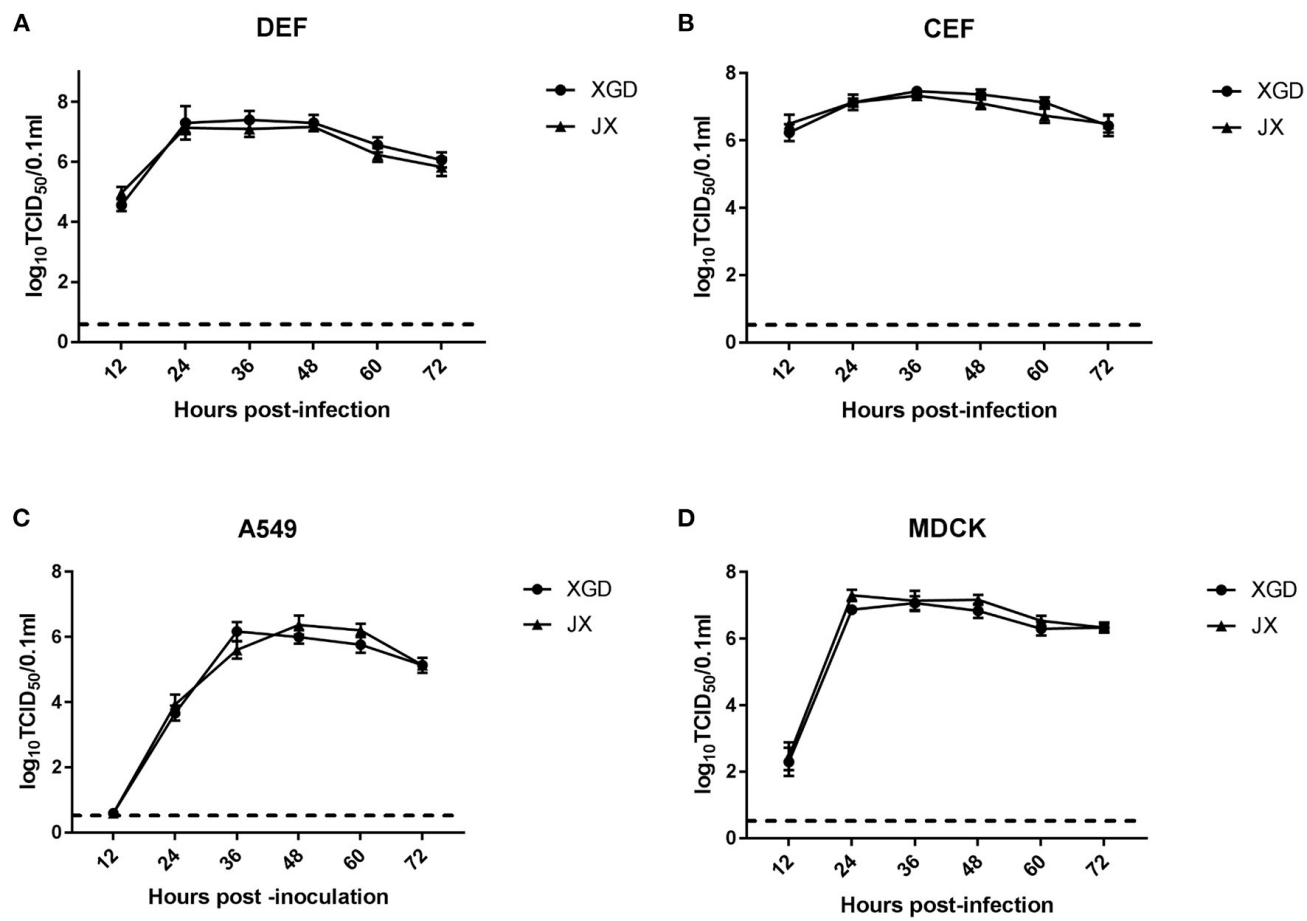
Growth kinetics of the XGD and JX strains of H5N6 *in vitro*. Confluent **(A)** duck embryo fibroblasts (DEF), **(B)** chicken embryo fibroblasts (CEF), **(C)** lung carcinoma cells (A549), and **(D)** Madin–Darby canine kidney (MDCK) cells infected with XGD or JX at a multiplicity of infection (MOI) of 0.01. Samples were collected at 12, 24, 36, 48, 60, and 72 h post-infection (hpi). Viral titers were determined using 50% tissue culture infective dose (TCID_50_) assays in MDCK cells. The dotted line represents the limit of detection of viral titers. Data are presented as the mean ± standard deviation of triplicate experiments. Statistical significance was analyzed using an independent sample *t*-test.

The growth kinetics of XGD and JX were compared in DEF, CEF, A549, and MDCK cells. XGD and JX showed similar replication at all time points in the four cell lines. Viral titers peaked at 12 hpi in DEF and CEF but showed delayed replication kinetics in A549 and MDCK cells (Figures 1A,B). Few viral titers were detected at 12 h post-infection (hpi) and peaked at 36 or 48 hpi in A549 cells (Figure 1C). For the MDCK cell line, moderate virus titers (10^2.7^ TCID_50_/0.1 ml) were detected at 12 hpi and the maximum viral titer kept from 24 to 48 hpi (Figure 1D). Therefore, no significant differences were observed in the growth kinetics of JX and XGD *in vitro*.

### Sialic Acid–Binding Activity Assay

**Figure 2:**
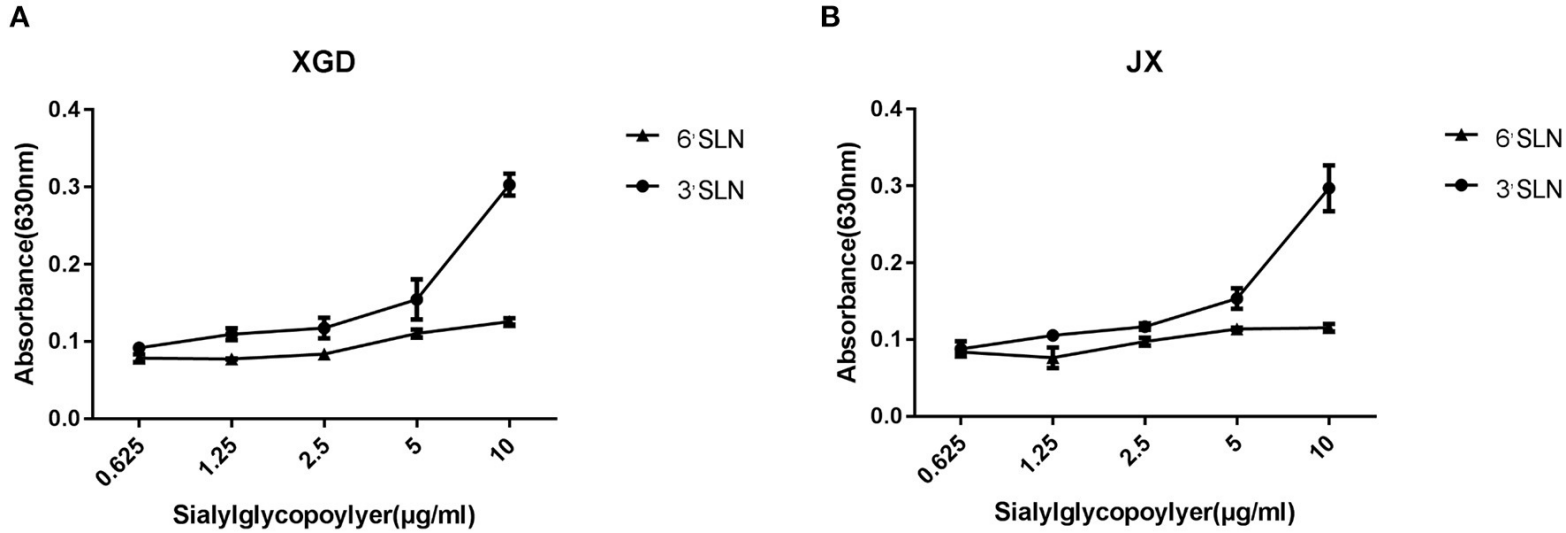
Sialic acid–binding activity assay of the JX and XGD strains of H5N6. The synthetic sialyl glycopolymers Neu5Aca2-3Galb1-4GlcNAcb-PAA-biotin (3′SLN) and Neu5Aca2-3Galb1-4GlcNAcb -PAA-biotin (6′SLN) were used to evaluate the receptor-binding properties of XGD **(A)** and JX **(B)** via solid-phase binding assays. Data are presented as the mean ± standard deviation of triplicate experiments. Error bars indicate one standard deviation.

Although avian cells are more conducive to the replication of IAV, H5N6 poses a considerable threat to human public health. AIVs preferentially bind to SAa2,3Gal receptors, whereas human influenza viruses bind to SAa2,6Gal receptors (20–22). To identify any changes in the receptor tropism of XGD and JX, we conducted a sialic acid–binding activity assay (Figure 2). The results showed that XGD and JX primarily bound to avian-like receptors (3'SLN) and rarely to human-like receptors (6'SLN). However, no significant differences in receptor-binding ability were identified between XGD and JX.

### Virulence of XGD and JX

**Figure 3:**
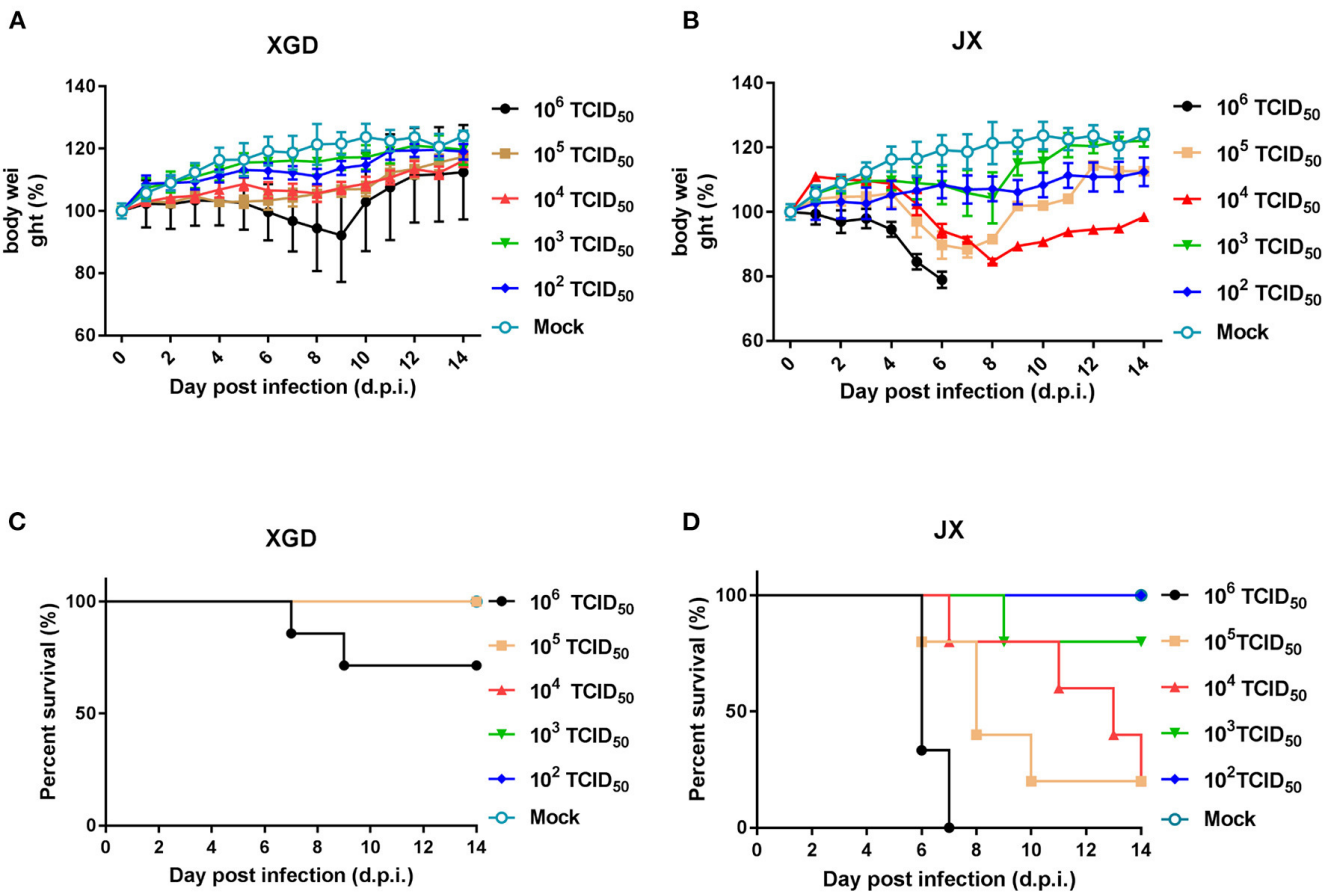
Weight loss and survival of mice infected with the XGD and JX strains of H5N6. Groups of mice (*n* = 5) were intranasally infected with 10-fold serial dilutions (10^2^-10^6^ TCID_50_) of XGD or JX. Control mice were inoculated with phosphate-buffered saline (PBS). The infected mice were observed to monitor weight loss **(A,B)** and survival **(C,D)** for 14 days post-infection (dpi). The mock infected mice are shared between group XGD and JX.

The virulence of XGD and JX was tested in groups of BALB/c mice infected intranasally with 10^2^-10^6^ TCID_50_ of each strain (Figure 3). Mice in control group were inoculated with PBS. In the XGD groups, only the highest dose (10^6^ TCID_50_) caused significant weight loss (Figure 3A). In JX groups, However, the group infected with 10^6^ TCID_50_ showed the highest weight loss (24.5%), at 7 dpi (Figure 3B), and 10^5^ TCID_50_ and 10^4^ TCID_50_ led to an average weight loss of 15.3 and 11.6%, respectively. Low doses (10^2^ and 10^3^ TCID_50_) did not cause any significant weight loss, but weight gain was slower than that in the control. Overall, weight loss was significantly higher in the JX groups than in the XGD groups. No deaths occurred in the other XGD groups, except in the mice infected with 10^6^ TCID_50_ virus. Based on the survival curves, the MLD_50_ was 10^3.7^ TCID_50_ for JX and 10^>6^ TCID50 for XGD (Figures 3C,D); thus, the former was more virulent.

**Figure 4:**
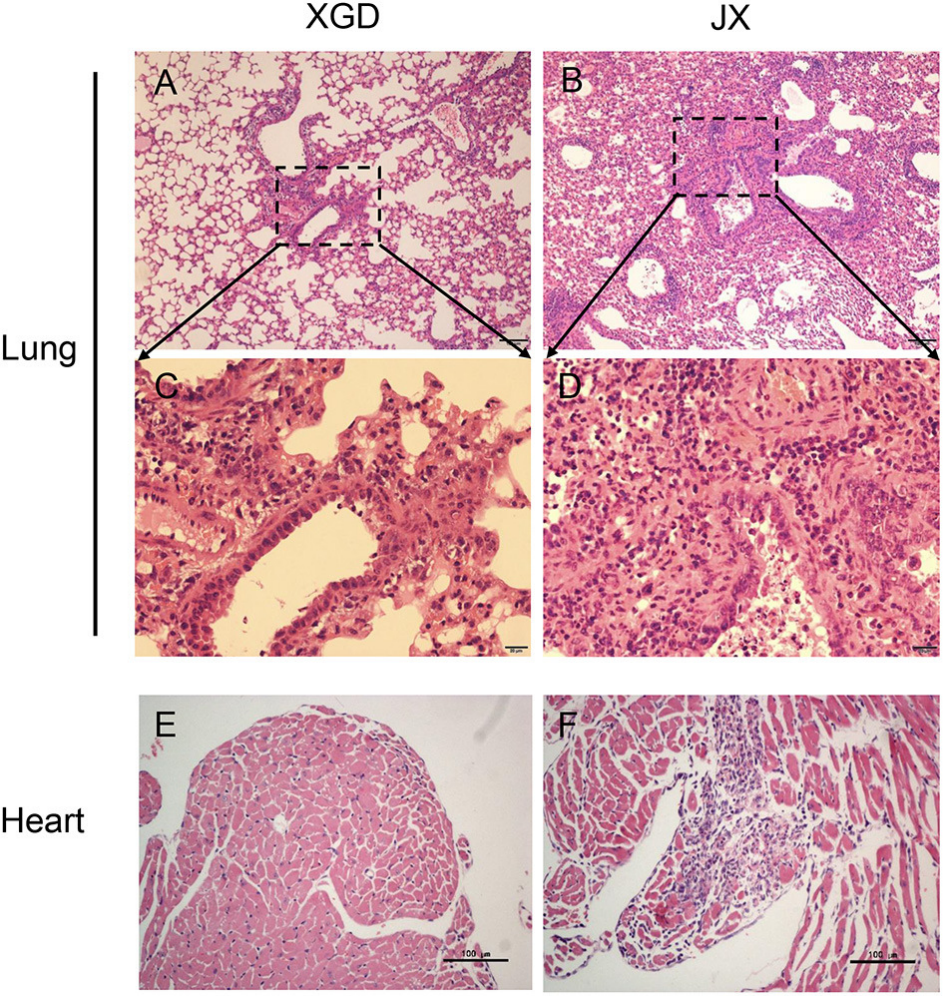
Histopathological analysis of the lungs and hearts of mice infected with the XGD and JX strains of H5N6. Groups of mice (*n* = 6) were intranasally infected with 10^5^ TCID_50_ of XGD or JX. Samples were collected at 5 days post-infection (dpi). Histopathological changes in mouse lungs **(A–D)** and hearts **(E,F)** induced by XGD or JX infection. Scale bar in **(A,B,E,F)** 100 μm; in **(C,D)** 20 μm.

To further assess the pathogenicity of XGD and JX, we performed a histopathological analysis (Figure 4). JX induced severe interstitial pneumonia characterized by alveolar lumens and bronchioles flooding with fibrin, erythrocytes, and inflammatory cells (Figures 4B,D). The infected mice exhibited influenza-related myocarditis characterized by inflammatory cell infiltration into the myocardium and myocardial damage (Figure 4F). XGD induced mild interstitial pneumonia characterized by thickened alveolar walls and inflammatory cell infiltration (Figures 4A,C). However, no signs of myocarditis were observed (Figure 4E).

**Figure 5:**
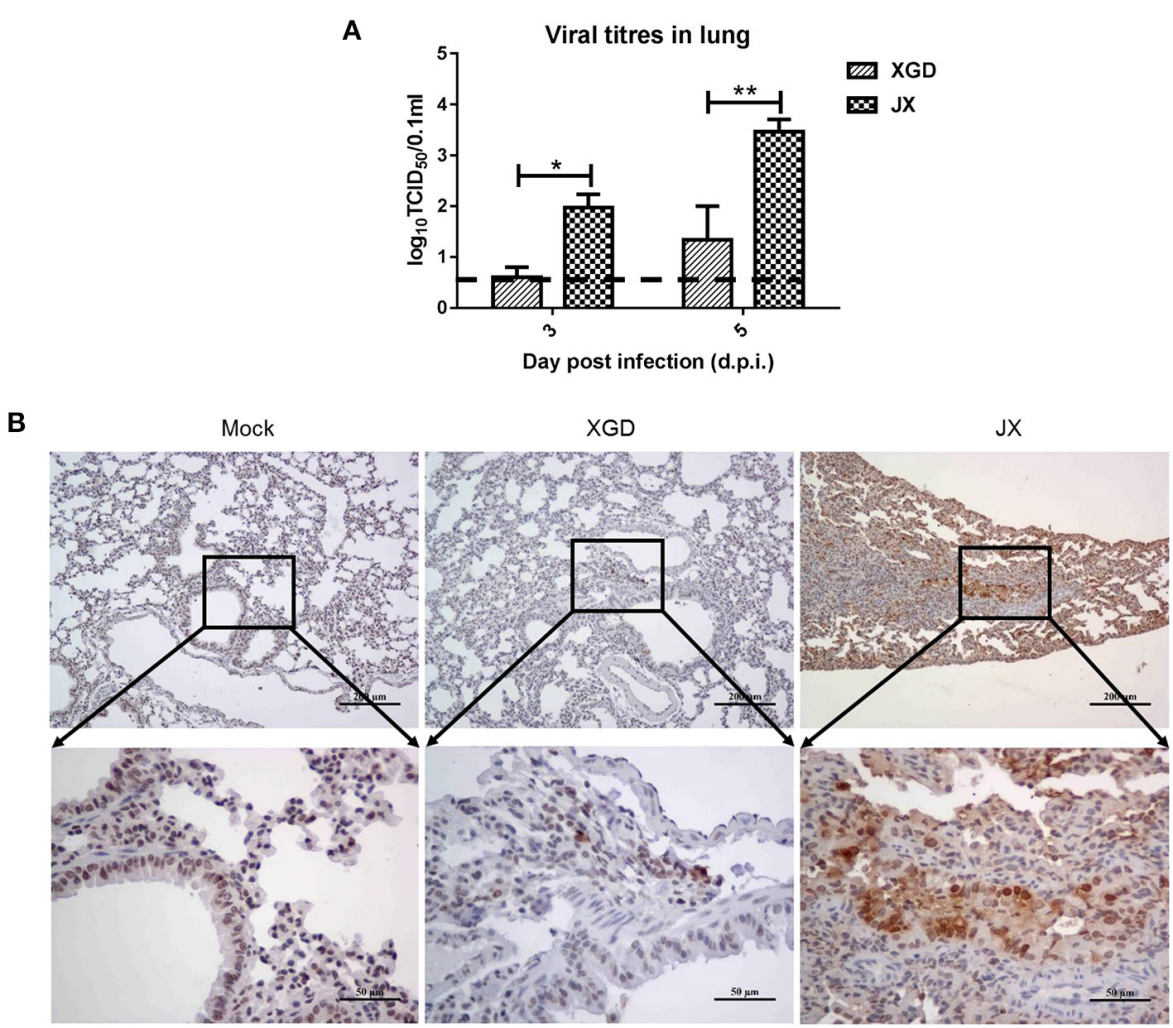
Viral loads and immunohistochemistry analysis of the lungs of mice infected with the XGD and JX strains of H5N6. Groups of mice (*n* = 6) were intranasally infected with 10^5^ TCID_50_ of XGD or JX. Control mice were inoculated with phosphate-buffered saline (PBS). Lungs were collected at 3 and 5 days post-infection (dpi). **(A)** XGD and JX titers in mouse lungs were calculated through TCID_50_ at 3 days post-infection (dpi) and 5 dpi. **(B)** Immunohistochemical detection of Nucleoprotein (NP) antigen in mouse lungs at 5 dpi. Scale bar in **(B):** the three images on the top, 200 μm; the three images at bottom: 50 μm.

A viral load analysis was conducted to assess JX and XGD replication in the lungs at 3 and 5 dpi (Figure 5A). JX titers were 10^2.0^ TCID_50_/0.1 mL at 3 dpi and 10^3.5^ TCID_50_/0.1 mL at 5 dpi, whereas XGD titers were 10^0.6^ TCID_50_/0.1 mL at 3 dpi and 10^1.3^ TCID_50_/0.1 mL at 5 dpi. Thus, the levels of XGD in mouse lungs were significantly lower than those of JX. Consistently, immunohistochemistry results showed a limited number of viral NP antigens in mouse lungs infected with XGD but a wide distribution of viral NP-positive bronchiolar epithelial and alveolar epithelial cells in mouse lungs infected with JX (Figure 5B). Overall, JX showed a higher pathogenicity than XGD in mice, causing substantial weight loss, severe lung and myocardial damage, and increased lethality.

### Pathogenicity of Recombinant Viruses

**Figure 6:**
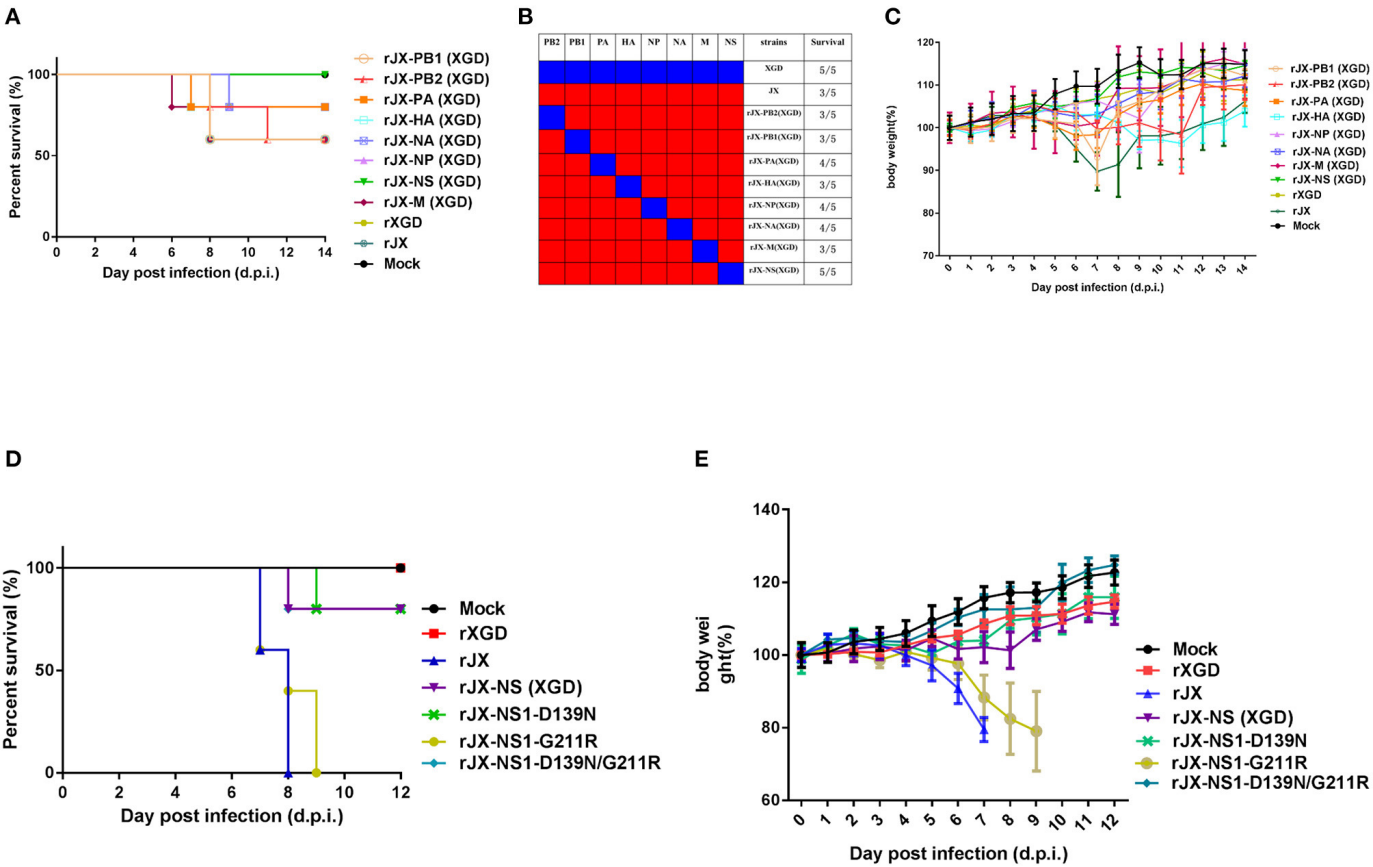
Pathogenicity of recombinant or mutant viruses. Groups of mice (*n* = 5) were intranasally infected with 10^3.7^ TCID_50_ of recombinant viruses. The infected mice were observed to monitor survival **(A,B)** and weight loss **(C)** for 14 days post-infection (d.p.i). Groups of mice (*n* = 5) were intranasally infected with 10^5^ TCID_50_ of NS1 mutant viruses. The infected mice were observed to monitor survival **(D)** and weight loss **(E)** for 12 dpi.

Recombinant viruses containing one segment of XGD rescued on a JX background were used to identify the viral segment contributing to high pathogenicity in mice. Mice (*n* = 5) were intranasally infected with 10^3.7^ TCID_50_ (the LD_50_ of JX) of each recombinant virus. One or two mice died in each group except the rJX-NS (XGD) and rXGD groups (Figures 6A,B). Moreover, these two groups showed no significant weight loss compared with the others (Figure 6C). Therefore, replacing NS of JX with that of XGD markedly reduced the virulence of JX. Sequencing analysis revealed 13 amino acid substitutions distributed among six proteins of XGD and JX (Table 1), of which two mutations were localized on NS1. Consequently, we generated the mutant viruses rJX-NS1-D139N/G211R, rJX-NS1-D139N, and rJX-NS1-G211R. Mice infected with rJX-NS1-D139N/G211R or rJX-NS1-D139N showed no significant weight loss, and all survived; however, rJX-NS1-G211R maintained the virulence of JX and led to high mortality (Figures 6D,E). Thus, 139D in NS1 might play an important role in JX virulence in mice.

**Table 1:** Amino acid variations in the JX and XGD strains of H5N6.

**Proteins**	**Positions**	**Mutations**	
		**XGD**	**JX**
PB2	140	K	R
	184	T	A
	249	G	E
	718	K	R
PA	360	M	L
HA	154	L	Q
	339	R	S
NP	374	I	M
NA	13	T	A
	171	A	T
	189	N	I
NS1	139	N	D
	211	R	G

## Discussion

Novel influenza viruses emerge through antigenic drift and shift. The low pathogenic avian influenza H9N2 subtype is widely spread throughout domestic and wild birds and might contribute to the generation of novel reassortants with altered pathogenicities and host ranges (23–26). Previous studies revealed that H5N6 contains the hemagglutinin of H5, the internal genes of H5, and the NA gene of avian H6N6 (27–29). Further research has suggested that the internal genes originated from the chicken H9N2/H7N9 gene pool (4, 30). In the present study, the XGD and JX strains of H5N6 were isolated from two sites that were approximately 100 km apart. All their genes originated from H5N1 and H6N6, and they had only 13 amino acid substitutions distributed among six proteins. Therefore, XGD and JX probably evolved from the same ancestral H5N6.

Avian influenza viruses have been identified in numerous wild and domestic bird species; however, aquatic birds are the natural viral reservoirs that contaminate the surrounding water environments (31–33). Most ducks infected with highly pathogenic avian IAV are asymptomatic, but infection with H5N6 of clade 2.3.4.4 can lead to mortality. Novel H5 viruses are also highly pathogenic to chickens (34). The strains XGD and JX of H5N6 are highly pathogenic to both ducks and chickens, but the former is more virulent to chickens than the latter. H5N6 has replaced H5N1 as the dominant IAV subtype in waterfowls. Moreover, avian H5N6 causes human infections with high mortality rates (4, 35, 36). A vaccination strategy has been implemented in the water poultry industry to mitigate outbreaks of novel H5 reassortants and prevent epidemics.

Influenza virus replication in host cells is mediated by a trimeric polymerase complex. Avian cells are more suitable for JX and XGD replication than mammalian cells because the polymerase complex needs to adapt in the latter through gene mutation or reassortment (35, 36). E627K and D701N in PB2 are the most common influenza virus adaptation mutations. Our study showed that JX and XGD replicated similarly in the same host cells, but JX showed stronger replication than XGD in mice.

Several viral proteins have been reported to contribute to the transmissibility, virulence, pandemic potential, and ability to cross IAV species barriers. NS1, a key virulence factor, antagonizes the IFN innate immune response by targeting various signal molecules. In addition, NS1 aids the shutdown of the host cellular machinery, promotes viral-only gene expression, and increases the infected cell survivability by activating PI3K and regulating the apoptotic response (37–41). It has been reported that the deletion of 80–84 amino acids in NS1 increases the pathogenic ability of H5N1 by hijacking the innate immune system of dendritic cells *in vivo* (42). Our study revealed that both JX and XGD had five amino acid deletions in NS1. According to Global Initiative on Sharing All Influenza Data (GISAID), of the 1256 NS1 sequences of H5N6 identified, 61.5% carry 139D, 26.9% carry 139N, and only a few carry the naturally acquired mutation 139G (https://platform.epicov.org/epi3/frontend#1e3e65). Moreover, we found that there were 37 human cases of infection with H5N6 virus in GISAID. To our surprise, 139D was located in all four human strains isolated in 2014, when the first case of human infection was reported. The patient infected with strain A/Sichuan/26221/2014 (H5N6) (EPI_ISL_163493) developed fever, severe pneumonia, leucopenia, and lymphopenia, septic shock and acute respiratory distress syndrome (ARDS), and died on day 10 after illness onset (29). However, the effect of the 139G mutation on NS1 function or on the virulence of H5N6 remains unclear.

The NS1 polypeptide comprises two distinct functional domains: an N-terminal RNA binding domain and a C-terminal effector domain (43). The 139D mutation is located in the latter, which predominantly mediates interactions with host-cell proteins. NS1 inhibits cellular pre-mRNA processing by binding to the 30 kDa subunit of cleavage and polyadenylation specificity factor (CPSF30) (44). Although 139D does not interact directly with CPSF30, it may be important for maintaining the NS1 structure needed for CPSF30 interactions (45).

In conclusion, we identified the key amino acid site 139D in the NS1 protein of JX, which might contribute to the high viral proliferative capacity and pathogenicity in mice. We propose that 139D in NS1 could serve as an indicator of avian influenza virus variants with potential public health risks. Still, more research is needed to elucidate the role of 139D in the high pathogenicity of H5N6 in mammals.

## Data Availability Statement

The original contributions presented in the study are included in the article/Supplementary Material, further inquiries can be directed to the corresponding author/s.

## Ethics Statement

All animal experiments were approved by the Research Ethics Committee, Huazhong Agricultural University, Hubei, China (HZAUMO-2016-022) and were performed in accordance with the Guidelines for the Care and Use of Laboratory Animals of the Research Ethics Committee, Huazhong Agricultural University, Hubei, China.

## Author Contributions

KH performed the majority of experiments and statistical analysis and wrote the manuscript. YZ, HM, PR, and XS participated in the protocol execution and data collection. ZZ and MJ provided the critical review of the manuscript. MJ was responsible for editing and revising the manuscript for the final version. All authors have read and approved the final version of the manuscript.

## Funding

This research was supported by the National Natural Science Foundation of China (31820103015 and 31702255).

## Conflict of Interest

The authors declare that the research was conducted in the absence of any commercial or financial relationships that could be construed as a potential conflict of interest.

## Publisher's Note

All claims expressed in this article are solely those of the authors and do not necessarily represent those of their affiliated organizations, or those of the publisher, the editors and the reviewers. Any product that may be evaluated in this article, or claim that may be made by its manufacturer, is not guaranteed or endorsed by the publisher.
