# Supplementary material for: 139D in NS1 Contributes to the Virulence of H5N6 Influenza Virus in Mice
Source: Front Vet Sci. 2022 Jan 21;8:808234. doi: 10.3389/fvets.2021.808234 (PMC8814418; doi:10.3389/fvets.2021.808234)
Supplement: Supplementary file 1 [file Table_1.docx]

**Supplementary materials**


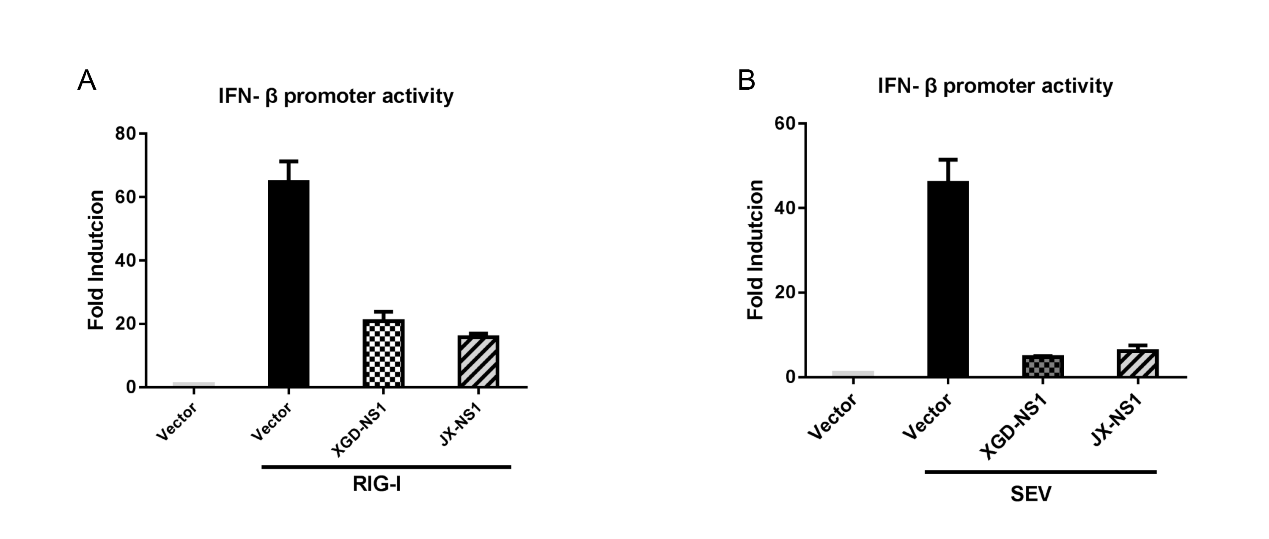


Figure S1. NS1 of XGD and JX inhibit IFN-β promoter activity induced by RIG-I or sendai virus (SEV). 293T cells were cotransfected with IFN-β-luc, the internal control pRL-TK and expression plasmid harboring NS1 (XGD or JX) or the empty vector. After 24 h, the cells were stimulated with RIG-I or SEV. Luciferase activity was measured at 24 h post-stimulation.
